# Unveiling key genetic determinants of charcoal rot resistance in soybean via genome-wide association studies

**DOI:** 10.3389/fpls.2025.1649397

**Published:** 2025-12-16

**Authors:** Vennampally Nataraj, Pawan Kumar Amrate, Milind B. Ratnaparkhe, Shivakumar Maranna, Laxman Singh Rajput, Nisha Agrawal, Rishiraj Raghuvanshi, Kriti Pathak, Saloni Mandloi, Salikram Mohare, Bhojaraja Naik K, Manoj K. Shrivastava, Giriraj Kumawat, Vangala Rajesh, Sanjay Gupta, Annapurna Chitikineni, Rajeev K. Varshney, K. H. Singh

**Affiliations:** 1 Indian Council of Agricultural Research (ICAR)-National Soybean Research Institute, Indore, Madhya Pradesh, India; 2 Jawaharlal Nehru Krishi Vishwa Vidyalaya, Jabalpur, Madhya Pradesh, India; 3 Indian Council of Agricultural Research (ICAR)-Central Arid Zone Research Institute, Jodhpur, Rajasthan, India; 4 Indian Council of Agricultural Research (ICAR)-Indian Institute of Seed Science and Technology, Bengaluru, Karnataka, India; 5 International Crops Research Institute for the Semi-Arid Tropics, Hyderabad, Telangana, India; 6 WA State Agricultural Biotechnology Centre, Centre for Crop and Food Innovation, Murdoch University, Perth, WA, Australia

**Keywords:** charcoal rot, genomics, oil seed, resistance, soybean

## Abstract

Charcoal rot is a soil- and seed-borne disease caused by a necrotrophic fungal pathogen—*Macrophomina phaseolina*. To understand the genetic architecture of resistance against it, a genome-wide association study (GWAS) was conducted based on a glasshouse experiment and a 3-year field experiment using 214 diverse soybean accessions. In a glasshouse experiment at the seedling stage, eight single-nucleotide polymorphisms (SNPs) were identified: one SNP each on chromosome (chr) 8 (*S8_16817767*), chr 10 (*S10_52066337*), chr 14 (*S14_50857981*), chr 15 (*S15_32620059*), chr 17 (*S17_1689021*), and chr 18 (*S18_9413708*), while two SNPs (*S16_34569104* and *S16_37878937*) were located on chr 16. In the case of the field experiment at the reproductive stage, 10 SNPs were identified: 1 SNP each on chr 12 (*S12_14977708*), chr 14 (*S14_51754926*), and chr 16 (*S16_33491560*), 2 SNPs each were identified on chr 6 (*S6_41109641* and *S6_41863847*) and chr 10 (*S10_40644409* and *S10_44768495*), while 3 SNPs (*S18_25004105, S18_55655188*, and *S18_56366541*) were located on chr 18. The SNP *S14_50857981* associated with seedling resistance and *S14_51754926* associated with adult plant resistance are present within the 1-Mb region and will be of immense importance for charcoal rot resistance breeding. The putative candidate gene analysis for identified SNPs revealed 23 genes with annotations associated with defense response pathways. Three genes encoding an NB-ARC domain associated with defense response were present near *S14_50857981*. The genotype PI 159923 was found to be resistant under both field and glasshouse conditions, and it will be employed as a parent in breeding for high-yielding charcoal rot-resistant genotypes. Our study provides new insights into charcoal rot resistance in soybean, identifying key SNPs and genes that can aid future breeding programs for developing climate-resilient crops.

## 1 Introduction

Soybean is a major oil seed crop with multi-faceted health benefits and industrial applications (Karikari et al., 2019). Though India ranks fifth in soybean production, its productivity is challenged by several biotic stresses. Among them, charcoal rot disease caused by *Macrophomina phaseolina* poses approximately 77% yield loss accounting for 39,200 metric tons (Wrather et al., 2010; Sharma et al., 2014). *M. phaseolina* is soil- and seed-borne in nature, and is a polyphagous necrotrophic fungal pathogen having a host range of approximately 500 plant species (Almeida et al., 2014; Iqbal and Mukhtar, 2020a, 2020b). This pathogen can attack soybean at any growth stage; seedlings, if infected, result in damping off, thereby affecting the plant stand. Aerial symptoms start to appear during the reproductive stage (R_4_–R_5_) (Fehr et al., 1971) where foliage starts to droop and gradually becomes yellow. The yellowing happens due to the blockage of xylem and phloem vessels by the fungal mycelia, which ultimately results in plant death (Iqbal et al., 2014; Amrate et al., 2023). The appearance of grayish-silver microsclerotia in the pith region of the stem and tap root is the diagnostic feature of this disease in soybean (Smith and Wyllie, 1999). Genomics and molecular breeding can be effective in mitigating soybean yield losses due to charcoal rot disease. Previous reports established the quantitative nature of resistance in soybean against this pathogen (Talukdar et al., 2009; Coser et al., 2017; Silva et al., 2019; Vinholes et al., 2019 and Zatybekov et al., 2023).

Genome-wide association studies (GWASs) are a potential tool in the genetic dissection of quantitative traits with high resolution. They use historical recombination in a diverse germplasm panel, evaluate a higher number of alleles per locus, and identify marker–trait associations in a short time (Susmitha et al., 2023). With the advancements in next-generation sequencing technology and single-nucleotide polymorphism (SNP) genotyping platforms, genomics is becoming effective in enhancing genetic gain in complex traits in crop plants. Genotype-by-sequencing (GBS) technology is a cost-effective high-throughput sequencing platform yielding simplified and uniform libraries, enabling its applicability in larger germplasm or breeding population sets (Bhat et al., 2016). This sequencing technology is being used in the identification of a large number of SNPs in a wide range of crop species to foster association mapping and genomic selection.

Association mapping relies on the linkage disequilibrium (LD) between the marker loci and functional gene governing the trait of interest. This LD can also result from the genetic relatedness in the form of population structure and kinship leading to false positives in GWASs (Kaler et al., 2020). To avoid it, several mixed linear models (MLMs) have been developed that take these two factors into consideration in identifying true associations between genetic variants and phenotypic polymorphism. However, these models are based on a single locus, and false-negative associations can occur due to overfitting (Kaler et al., 2020). To minimize this problem, several multi-locus mixed models (MLMMs) have been developed and utilized. FarmCPU (fixed and random model circulating probability unification) (Liu et al., 2016) and BLINK (Bayesian information and LD iteratively nested keyway) (Huang et al., 2019) are the two MLMMs predominantly used in GWASs across crop species including soybean (Xiong et al., 2023; Bhat et al., 2022; Yu et al., 2022). In a simulation study in soybean and maize, FarmCPU outperformed seven other models in identifying significant and true marker–trait associations (Kaler et al., 2020). In soybean, GWAS has been employed in understanding the genetic architecture and identifying loci/genes governing several traits like grain yield (Priyanatha et al., 2022), quality traits (He et al., 2021; Malle et al., 2020), abiotic stress tolerance (Sharmin et al., 2021), nutrient use efficiency (Mamidi et al., 2014; Wang et al., 2024), and *Phytophthora* resistance (You et al., 2024).

Given the importance of this disease in soybean, only a few attempts were made in understanding the genetics of charcoal rot resistance and in identifying the potential resistance donors (Coser et al., 2017; Vinholes et al., 2019; Zatybekov et al., 2023 and Amrate et al., 2023). Therefore, the current study was carried out (1) to identify charcoal rot resistance donors under glasshouse conditions and sick plot conditions, and (2) to identify SNP loci, haplotypes, and the putative candidate genes governing charcoal rot resistance in soybean.

## 2 Material and methods

### 2.1 Plant material

The association mapping panel (*N* = 214) used in the current study encompasses a diverse set of genotypes including exotic accessions (127), indigenous accessions (5), breeding lines (34), mutant lines (4), varieties (40), and unknown sources (4) (**Supplementary File**).

### 2.2 Phenotyping of soybean germplasm accessions for charcoal rot resistance at the seedling stage

**Figure 1:**
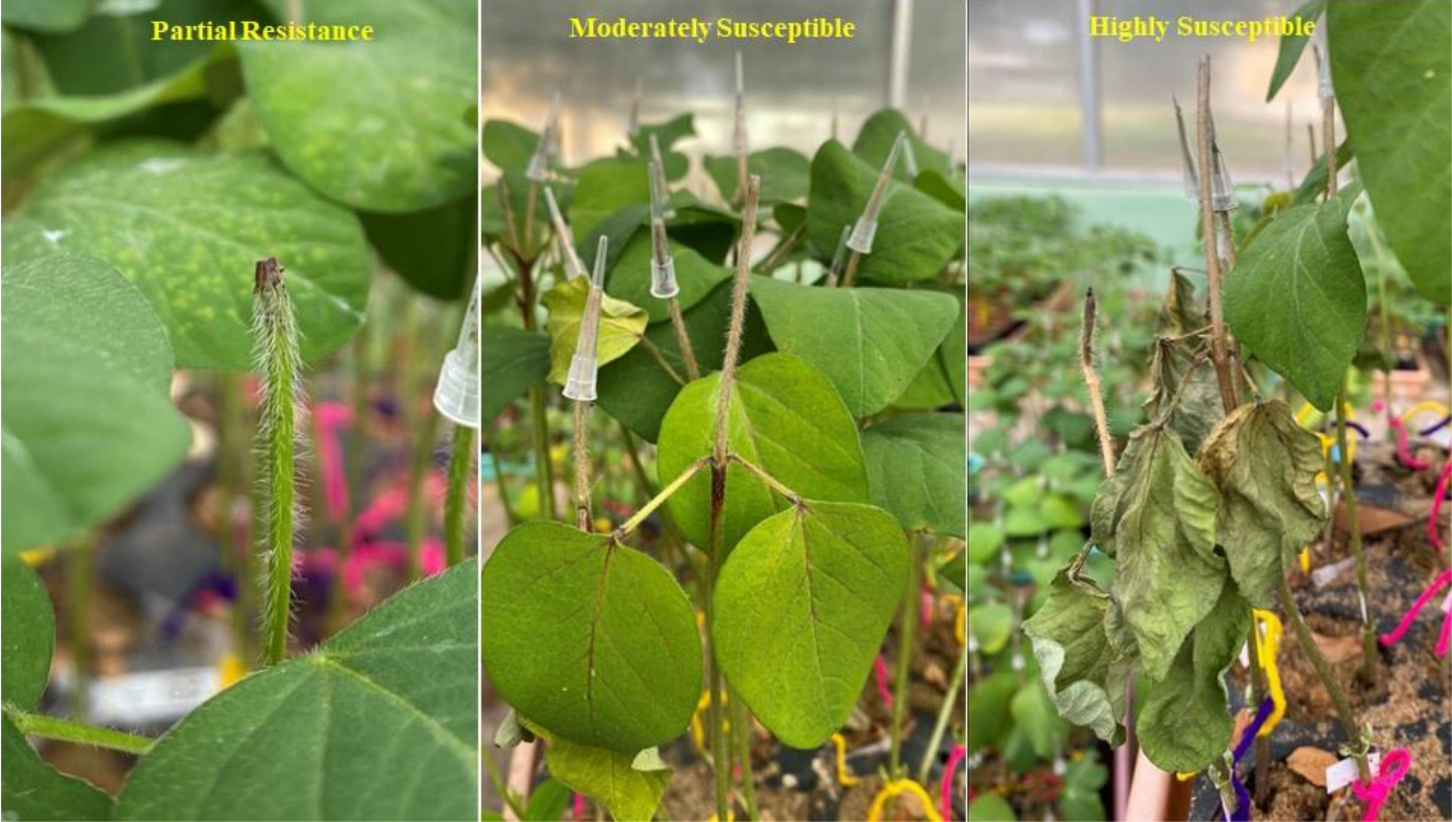
Phenotyping of soybean germplasm accessions for charcoal rot resistance at the seedling stage through an artificial inoculation method.

The GWAS panel was phenotyped for charcoal rot resistance at the seedling stage through the cut stem inoculation technique (Twizeyimana et al., 2012). After fulfilling Kotch’s postulates, the pathogen (Jabalpur isolate—NCBI ID: OR467498) re-isolated from a susceptible genotype was used for artificial screening. The glasshouse was maintained at 28 ± 2 °C day/night temperature and at 65% relative humidity. A randomized complete block design (RCBD) was followed by replicating each genotype four times. Using a sharp sterilized lazar blade, seedlings at their V_2_ growth stage (completely unrolled leaf at the first node above the unifoliolate node) (Fehr et al., 1971) were cut horizontally 4 cm above the unifoliate node. A disc full of actively growing mycelia from a 4-day-old fungal culture was collected with the help of the broad end of the pipette tip (10 μL) and was kept and retained on the cut portion of the stem tip. The length of the stem necrosis (in centimeters) that progressed linearly was measured 5, 10, and 15 days after inoculation (**Figure 1**). Disease resistance evaluation was based on the stem necrosis length 15 days after inoculation and the area under disease progress curve (AUDPC) (Shaner and Finney, 1977).

### 2.3 Phenotyping of soybean germplasm accessions for charcoal rot resistance at the adult plant stage

**Figure 2:**
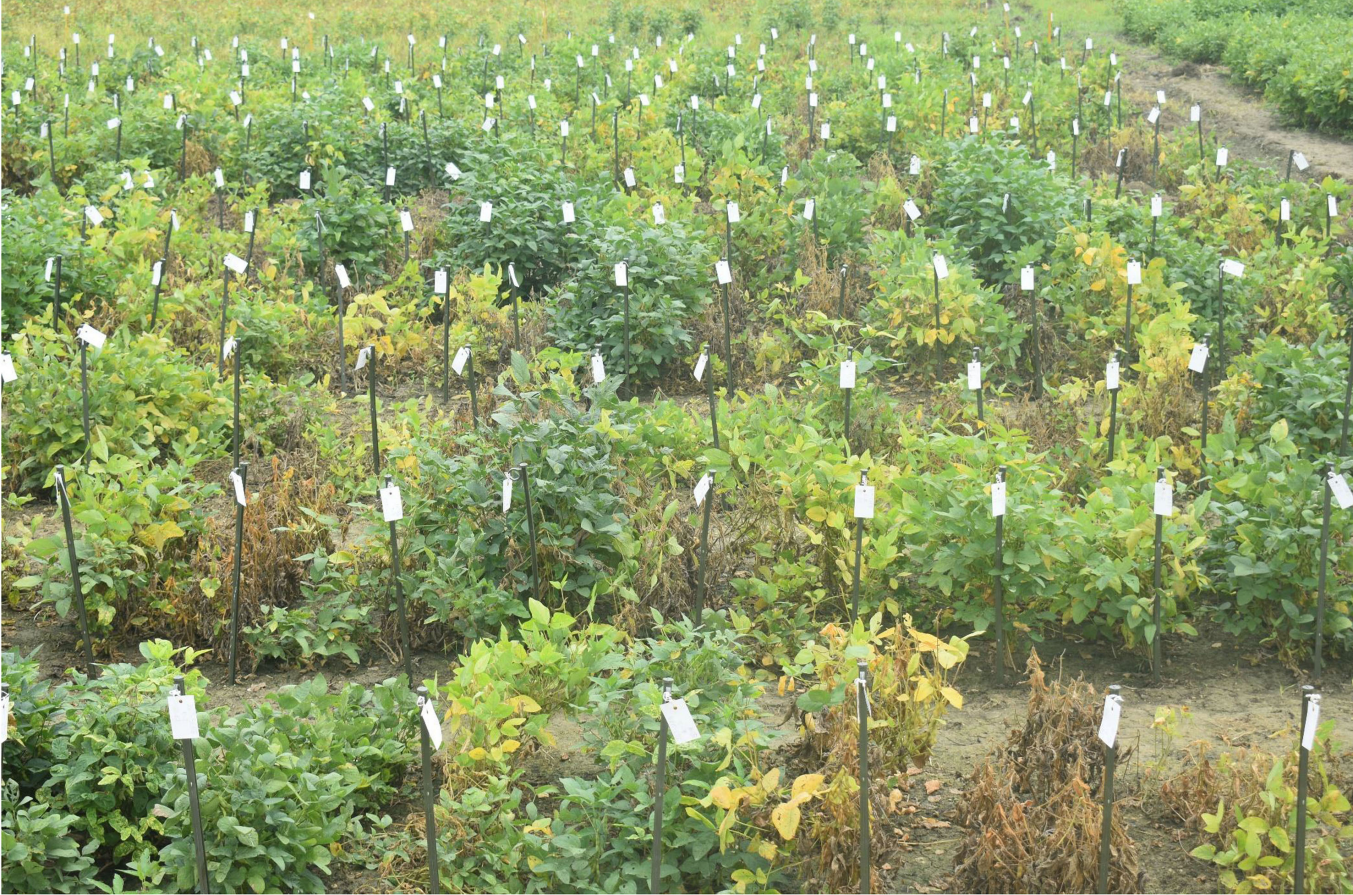
Phenotyping of soybean germplasm accessions for charcoal rot resistance at the adult plant stage under sick plot conditions.

**Figure 3:**
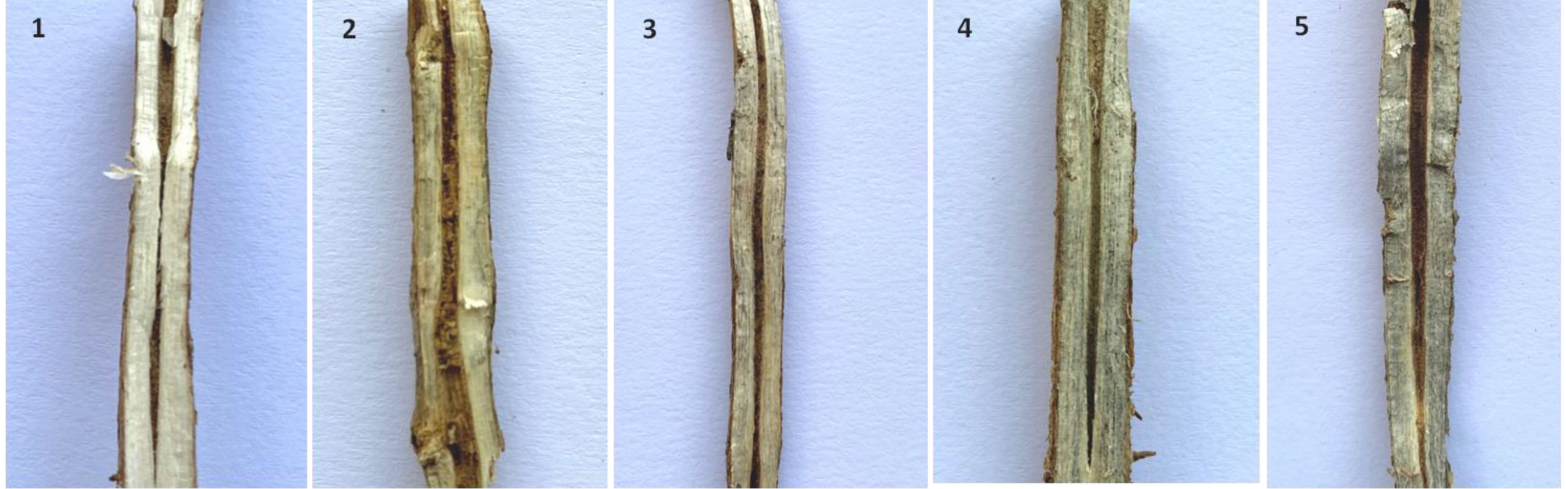
Phenotyping of germplasm accessions through the root stem severity (RSS) index. Disease rating scale (1–5) was as per Mengistu et al. (2007).

The same set of genotypes was evaluated for charcoal rot resistance under sick-plot conditions at Jawaharlal Nehru Krishi Vishwavidyalaya, Jabalpur, India, for three consecutive years: 2021, 2022, and 2023 (**Figure 2**). The experimental design followed was RCBD replicating each genotype three times. Seeds were hand sown in a 1-m row with 45 cm row-to-row distance and 5 cm plant-to-plant distance within the row. Two susceptible checks (JS 95–60 and JS 93-05) were sown after 10 rows every time so as to ensure uniform disease occurrence and no disease escape. Disease resistance evaluation was based on percent disease incidence (PDI) at the R_7_ stage (physiological maturity), AUDPC, and root stem severity (RSS) index. After 60 days of sowing, PDI was measured at an interval of every 7 days for 6 weeks and AUDPC was calculated as per the above section. For RSS, five randomly pre-tagged plants in each line were uprooted gently at the R_7_–R_8_ stage (physiological maturity–harvest maturity). Their stem and taproot portion was longitudinally split with a sharp knife and the microsclerotial density in the pith region was scored based on a 1–5 scale (Mengistu et al., 2007) (**Figure 3**).

### 2.4 Genotyping and SNP quality control

GBS-derived SNP data of the 214 soybean accessions used in this study were obtained from study of Raghuvanshi et al. (2025). Briefly, GBS-derived FASTQ files were processed and then mapped against the soybean genome Glyma.Lee_v2.0 (Legumepedia database), and SNPs were called using the Fast-GBS.v2 pipeline (Torkamaneh et al., 2020c). K-nearest neighbor (KNN) imputations were performed in TASSEL software to fill missing genotype data. SNPs were filtered for minor allele frequency (MAF) < 0.05 and missing rate >10%, and finally a total of 66,976 SNPs distributed all over 20 chromosomes were used for association studies.

### 2.5 Genetic diversity and population structure analysis

A neighbor-joining tree, principal component analysis (PCA), and an LD decay plot of 214 soybean accessions using SNPs were generated using the GAPIT package (https://www.maizegenetics.net/gapit) implemented in R. Population structure was developed using STRUCTURE software (https://web.stanford.edu/group/pritchardlab/structure.html).

### 2.6 Genome-wide association studies

The analysis involved 214 diverse soybean germplasm accessions to study traits associated with charcoal rot resistance across 3 years (2021–2023). The association analysis was performed using two models—FarmCPU (Liu et al., 2016) and BLINK (Huang et al., 2019)—using the R package “GAPIT3” (Wang and Zhang, 2021). The first two principal components were included as covariates in both models. Significant SNPs were identified using an empirical significance threshold value of −Log10 *p* ≥ 4.0, equivalent to a *p*-value ≤ 0.0001, which has previously been reported to be appropriate for complex traits and has been used in previous studies (Chamarthi et al., 2021). Furthermore, to check false discovery rate (FDR), Bonferroni threshold was calculated by dividing probability level (0.05) with the total number of SNPs used, which yielded a cutoff of 7.46 e−7 (Gao et al., 2010). Those SNPs with the *p*-value above the cutoff were considered as “significant SNPs” while those below the cutoff were considered as “suggestive SNPs”. Manhattan plots illustrated significant markers, while quantile–quantile (Q–Q) plots compared expected versus observed *p*-value distributions (on a −log10 scale).

### 2.7 Identification of putative candidate genes

The SNPs (with *p* > 0.0001) identified for multiple resistance traits were further used to analyze the putative candidate gene annotation from genomic regions 200 kb upstream and downstream of these SNPs (totaling 400 kb). Gene models within these regions were downloaded, and annotation data were obtained from the corresponding locations on the Williams 82 reference genome assembly Wm82.a2.v1 from SoyBase (www.soybase.org). The genes were narrowed down by gene ontology (GO)-based biological process descriptions related to defense response and antifungal activity and PFAM descriptions for disease resistance genes.

### 2.8 Haplotype analysis

Haplotypes were analyzed within the LD region by using DnaSP software version 5.10 (http://www.ub.edu/dnasp/index_v5.html). To evaluate the effect of the haplotypes containing different combinations of alleles in the SNP loci associated with the putative candidate gene, the genotypes were grouped according to their haplotype in the SNP. Genotypes were grouped into independent clusters according to their specific SNP alleles, and means were compared using Tukey’s HSD (honestly significant difference) test. The average of the AUDPC in each group was calculated and represented graphically. Since all other genome regions remained randomly represented in each group, the difference in the averages of each group is a function of the fixed haplotypes in each group. Furthermore, the “*t*-test” was performed to determine significant differences in the mean of the AUDPC in two groups with allelic difference at the peak SNP, S14_51754926.

## 3 Results

### 3.1 SNP marker distribution across the 20 chromosomes

After filtration, a total of 66,976 polymorphic SNPs (MAF < 0.05) were retained for analysis. The highest number of SNPs was located on chromosome 18 (6,275), followed by chromosome 16 (4,326), chromosome 6 (4,237), and chromosome 13 (4,116). The least number of SNPs was located on chromosome 12 (2,071), followed by chromosome 19 (2,365) and chromosome 1 (2,442).

### 3.2 Population structure, genetic diversity, and linkage disequilibrium

**Figure 4:**
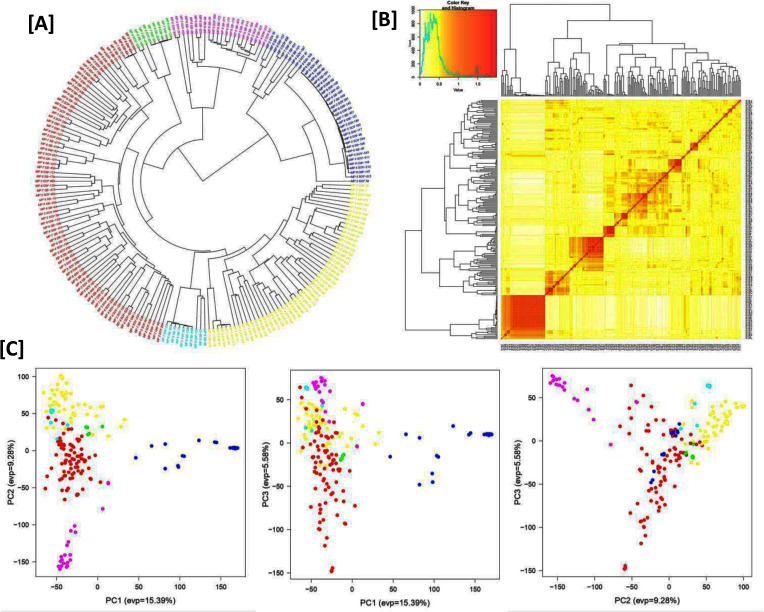
Genetic diversity and relatedness of the soybean germplasm accessions. **(A)** Neighbor-joining tree constructed using 66,976 SNP data. A total of six different clades were observed in our GWAS panel. **(B)** A kinship plot. A heat map of the values in the kinship matrix, showing the level of relatedness among the GWAS panel (the darker area showing a highly related genotype and also from a different origin with the rest of the population). **(C)** 2D principal component analysis (PCA) for the entire GWAS panel derived from SNP data.

Population structure analysis revealed that Δ*K* was highest when *K* was set at six (**Figures 4A–C**), indicating the grouping of the 214 germplasm accessions into six distinct subpopulations. This stratification was also supported by the neighbor-joining phylogenetic tree, which displayed six clades (**Figure 4A**), and was consistent with the clustering observed in the PCA (**Figure 4C**). Additionally, LD analysis showed that the average genome-wide LD for the diversity panel was *r*² = 0.471.

### 3.3 Phenotypic evaluation under glasshouse conditions

**Figure 5:**
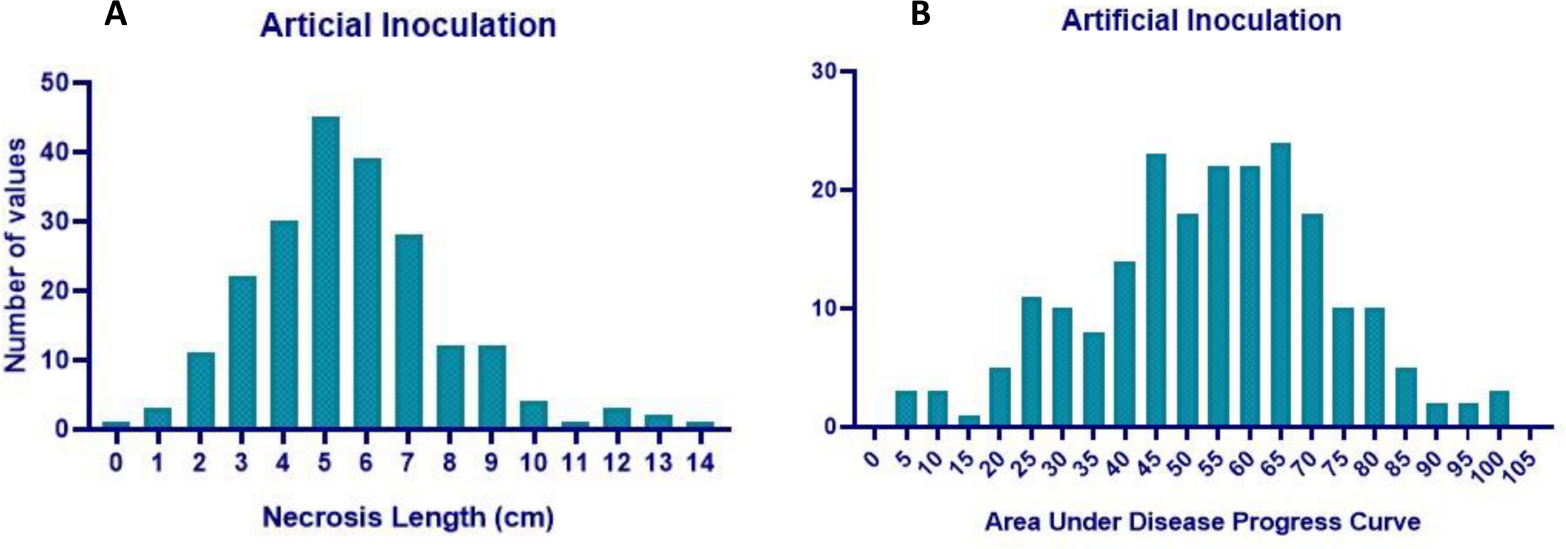
Histogram of necrosis length (cm) and AUDPC under glasshouse conditions.

Analysis of variance (ANOVA) indicated a significant genotypic effect for AUDPC and necrosis length. Mean AUDPC was 53.82, ranging from 4.62 to 102.27, while mean necrosis length was 5.58 cm with a range of 0.48–13.80 cm (**Table 1**). The top 10 best genotypes in the case of AUDPC were MACS 1520 (4.62), IC 15759 (5.00), B 1667 (6.93), Young (8.27), Bragg (8.79), MACS 13 (12.30), PI 159923 (13.22), PK 262 (17.97), EC 251498 (18.56), and TGX 86-24-1D (20.68). The top 10 best genotypes in the case of necrosis length were MACS 1520 (0.48 cm), Bragg (0.95 cm), IC 15759 (1.05 cm), PI 159923 (1.25 cm), EC 251498 (1.72 cm), TGX 86-24–1 D (1.95 cm), PK 262 (1.95 cm), Young (2.04 cm), B 1667 (2.10 cm), and EC 457305 (2.15 cm) (**Table 2**) The frequency distribution of the panel for necrosis length and AUDPC is depicted in **Figure 5**.

**Table 1:** Analysis of variance for the area under disease progress curve and necrosis length under the artificial inoculation experiment.

Source of variation	DF	*F* calculated	
		AUDPC	Necrosis length
Genotype	213	7.37^***^	5.45^***^
Replication	3	6.90^***^	3.67^**^
Residual	639	**-**	**-**
Mean		53.82	5.58
Range		4.62–102.27	0.48–13.80

DF, degrees of freedom; AUDPC, area under the disease progress curve.

^***^Significance at *p* < 0.001, ^**^Significance at *p* < 0.01.

**Table 2:** Trait-wise top 10 best genotypes under the glasshouse study.

Genotype	AUDPC^#^	Genotype	Necrosis length^#^
MACS 1520	4.62^a^	MACS 1520	0.48^a^
IC 15759	5.00^a^	Bragg	0.95^ab^
B 1667	6.93^ab^	IC 15759	1.05^abc^
Young	8.27^abc^	PI 159923	1.25^a-d^
Bragg	8.79^a-d^	EC 251498	1.72^a-e^
MACS 13	12.30^a-e^	TGX 86-24–1 D	1.95^a-f^
PI 159923	13.22^a-f^	PK 262	1.95^a-f^
PK 262	17.97^a-g^	Young	2.04^a-g^
EC 251498	18.56^a-h^	B 1667	2.10^a-h^
TGX 86-24–1 D	20.68^a-i^	PI 567186	2.15^a-i^

**^#^**Least significant difference (LSD) test (*p* < 0.05). AUDPC, Areas Under Disease Progress Curve. Means that do not share a common alphabetic letter are significantly different from each other.

### 3.4 Phenotypic evaluation under sick plot conditions

Across 3 years, the two checks (JS 95–60 and JS 93-05) showed susceptible disease reaction, indicating sufficient and uniform disease pressure in the sick plot. Pooled ANOVA revealed a significant genotype and environment effect and a significant genotype × environment interaction (*p* < 0.0001) (**Table 3**). The mean PDI was 53.83, 52.51, and 33.40 during 2021, 2022, and 2023, respectively (**Table 4**).

**Table 3:** Pooled analysis of variance for PDI, AUDPC, and RSS during 2021, 2022, and 2023.

Source of variation	DF	*F* calculated		
		PDI	AUDPC	RSS index
Genotype	213	18.79^***^	16.82^***^	12.51^***^
Environment	2	267.37^***^	349.66^***^	173.27^***^
Genotype × Environment	426	3.26^***^	2.90^***^	2.48^***^
Residuals	1,278	–	–	–

^***^Significance at *p* < 0.001.

**Table 4:** Year-wise descriptive statistics on different traits evaluated under sick plot conditions.

Traits	Year	Min	Max	Mean
PDI %	2021	0.00	100.00	53.83
	2022	0.00	100.00	52.51
	2023	0.00	93.33	33.40
RSS	2021	1.13	4.66	3.07
	2022	1.06	4.20	2.72
	2023	1.05	4.33	2.50
AUPDC	2021	0.00	2,090.75	929.02
	2022	0.00	2,430.78	944.18
	2023	0.00	1,627.77	481.64

**Figure 6:**
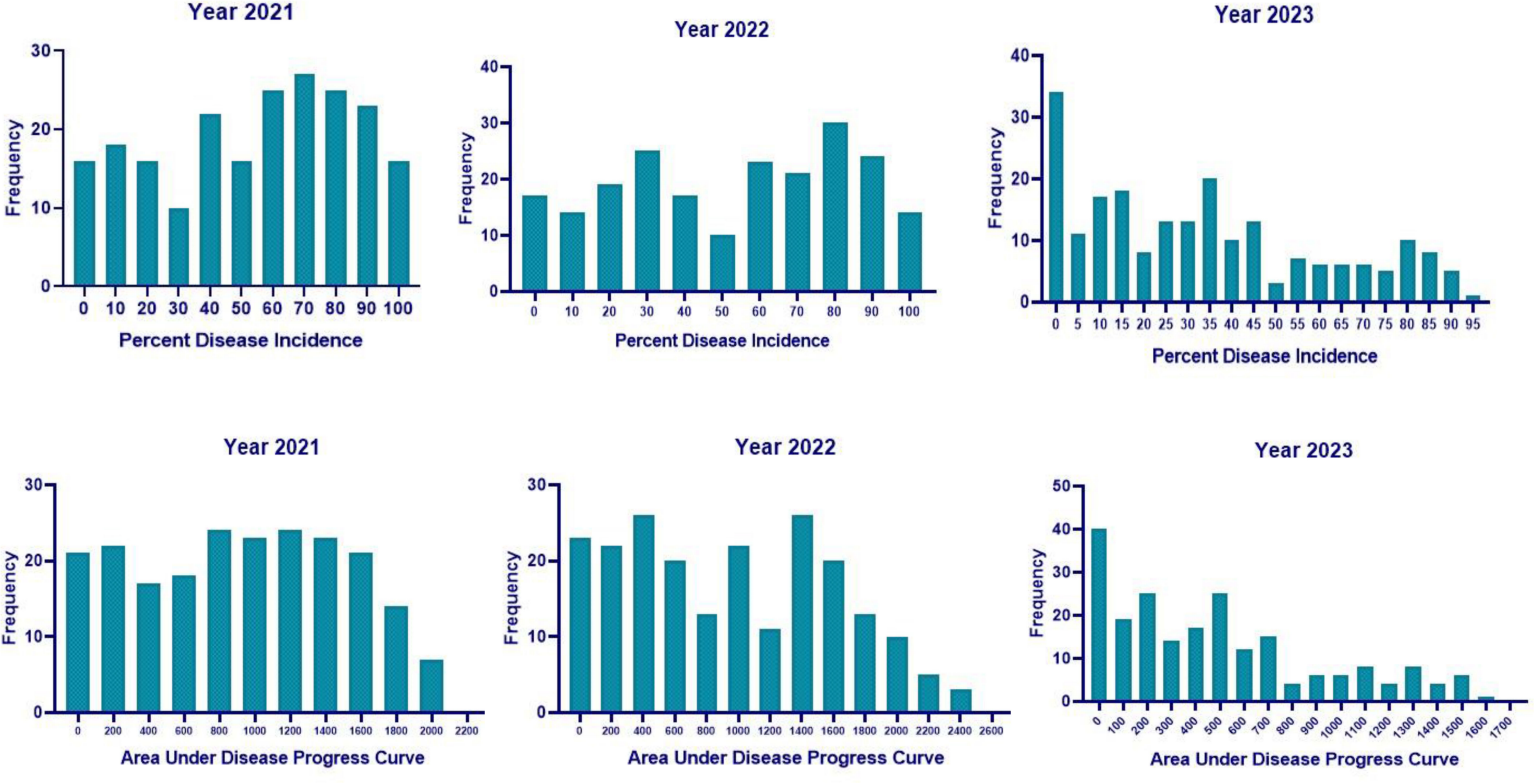
Histogram of PDI and AUDPC in 2021, 2022, and 2023 under sick plot conditions.

**Figure 7:**
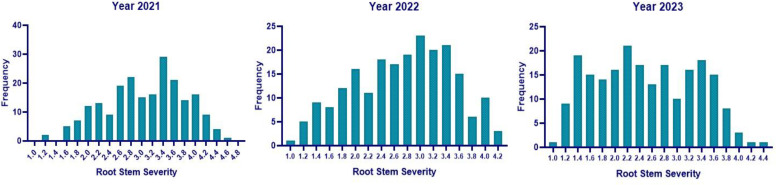
Histogram of RSS in 2021, 2022, and 2023 under sick plot conditions.

The mean RSS index was 3.07, 2.72, and 2.50 during 2021, 2022, and 2023, respectively. During 2021, the mean AUDPC was 929.02, while it was 944.18 and 481.64 during 2022 and 2023, respectively (**Table 4**). The 10 best-performing genotypes with respect to PDI were PI 159923 (0.0%), AGS 25 (0.69%), EC 602288 (1.58%), EC 393231 (1.85%), Lesoy 273 (1.85%), Pusa 16 (3.09%), NRC 2396 (3.17%), AMS 100-39 (3.61%), BRG 1 (3.80%), and EC 457516 (3.93%). In the case of AUDPC, the 10 best-performing genotypes were PI 159923 (0.00), AGS 25 (2.43), Lesoy 273 (6.48), EC 393231 (32.40), EC 602288 (38.88), NRC 2396 (44.44), Pusa 16 (50.48), PI 371609 (52.77), AMS 100-39 (54.62), and MAUS 71 (56.81). Genotypes EC 393231 (1.31), PI 159923 (1.35), BRG 1 (1.48), AGS 25 (1.51), JS 20-73 (1.51), NRC 2396 (1.55), JS 20-76 (1.55), EC 602288 (1.55), MAUS 71 (1.57), and Pusa 16 (1.57) were found to have the least RSS score (**Table 5**). The frequency distribution of the panel for PDI and AUDPC is depicted in **Figure 6** and RSS is depicted in **Figure 7**.

**Table 5:** Trait-wise 10 best-performing genotypes across 3 years.

Genotype	PDI^#^	Genotype	AUDPC^#^	Genotype	RSS^#^
PI 159923	0.00^a^	PI 159923	0.00^a^	EC 393231	1.31^a^
AGS 25	0.69 ^ab^	AGS 25	2.43^a^	PI 159923	1.35^ab^
EC 602288	1.58^abc^	Lesoy273	6.48^a^	BRG 1	1.48^abc^
EC 393231	1.85^abc^	EC 393231	32.40^ab^	AGS 25	1.51^a-d^
Lesoy 273	1.85^abc^	EC 602288	38.88^ab^	JS 20-73	1.51^a-d^
Pusa 16	3.09 ^a-e^	NRC 2396	44.44^abc^	NRC 2396	1.55 ^a-e^
NRC 2396	3.17 ^a-e^	Pusa 16	50.48^abc^	JS 20-76	1.55 ^a-e^
AMS 100-39	3.61 ^a-e^	PI 371609	52.77^a-d^	EC 602288	1.55 ^a-e^
BRG 1	3.80 ^a-e^	AMS 100-39	54.62 ^a-d^	MAUS 71	1.57 ^a-f^
EC 457516	3.93 ^a-e^	MAUS 71	56.81 ^a-d^	Pusa 16	1.57 ^a-f^

**^#^**Least significant difference (LSD) test (*p* < 0.05). AUDPC, Areas Under Disease Progress Curve; PDI, Percent Disease Incidence; RSS, Root Stem Severity. Means that do not share a common alphabetic letter are significantly different from each other.

### 3.5 GWAS analysis and prediction of putative candidate genes

**Figure 8:**
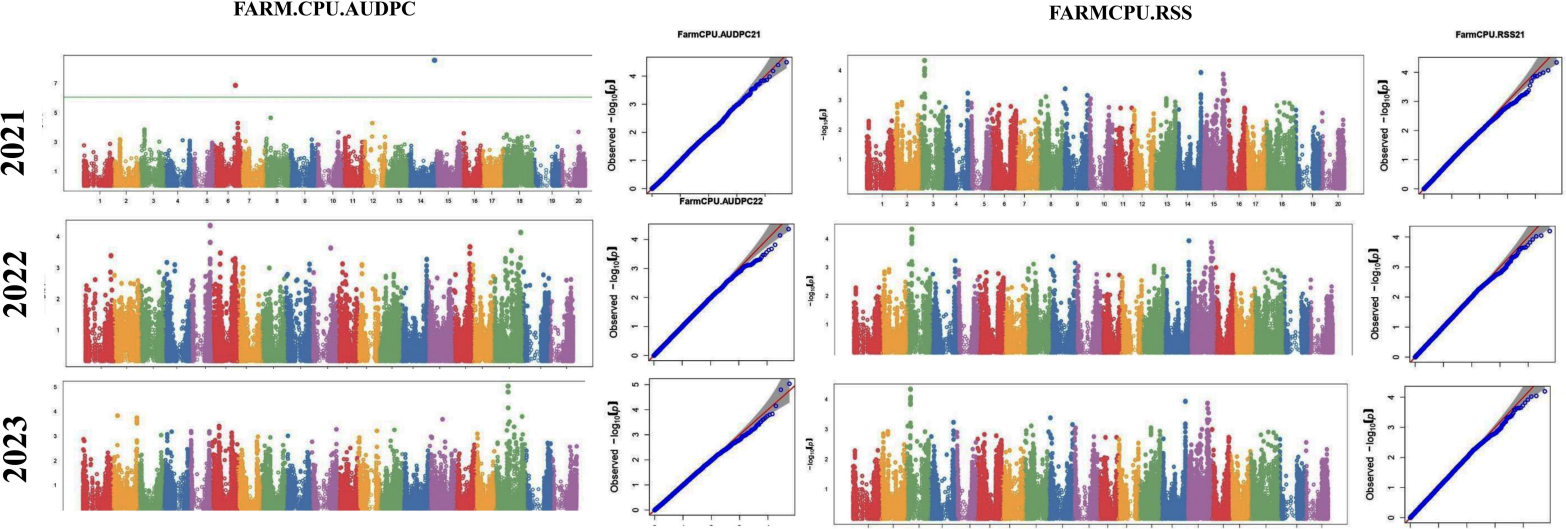
Manhattan plots (left) and quantile–quantile (Q–Q plots) (right) of the genome-wide association results for stem necrosis and AUDPC generated through FarmCPU and BLINK.

**Figure 9:**
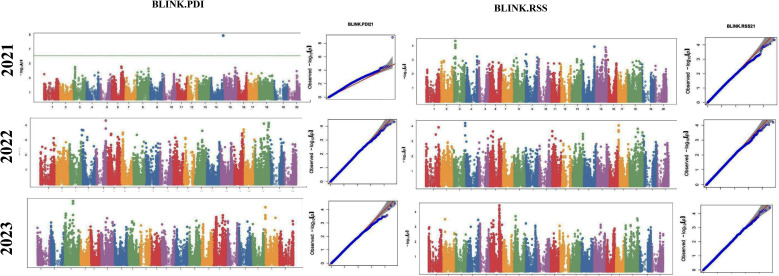
Manhattan plots (left) and quantile–quantile (Q–Q plots) (right) of the genome-wide association results for AUDPC generated through FarmCPU and BLINK in 2021, 2022, and 2023.

**Figure 10:**
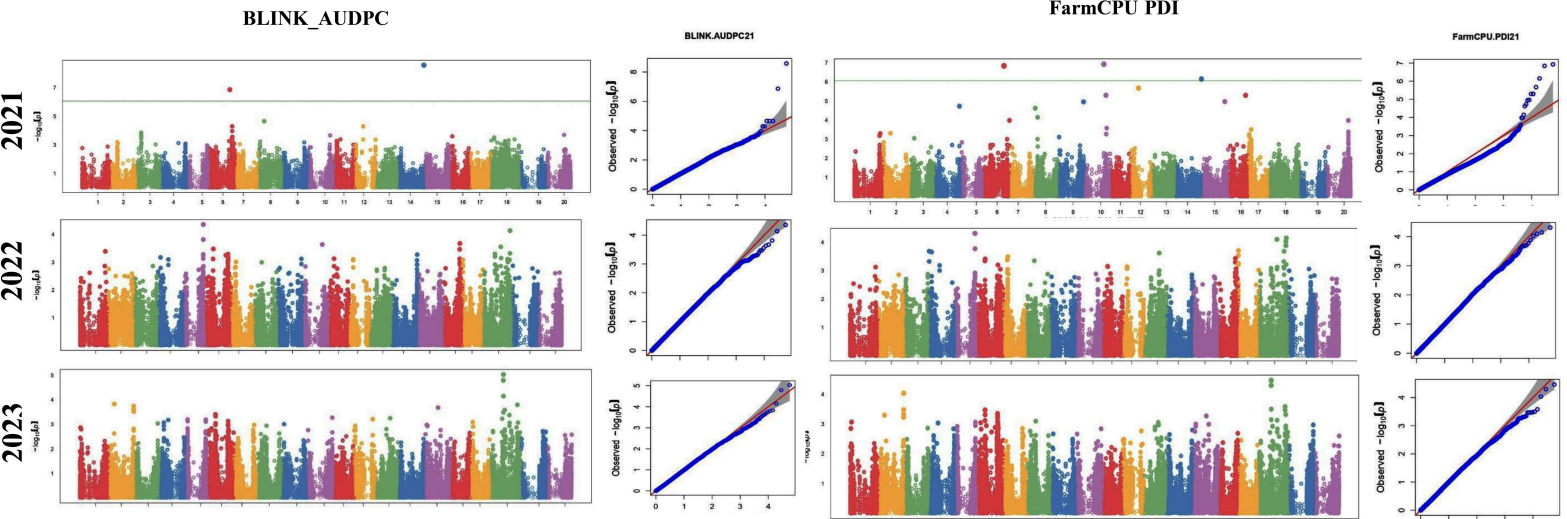
Manhattan plots (left) and quantile–quantile (Q–Q plots) (right) of the genome-wide association results for PDI generated through FarmCPU and AUDPC through BLINK in 2021, 2022, and 2023.

**Figure 11:**
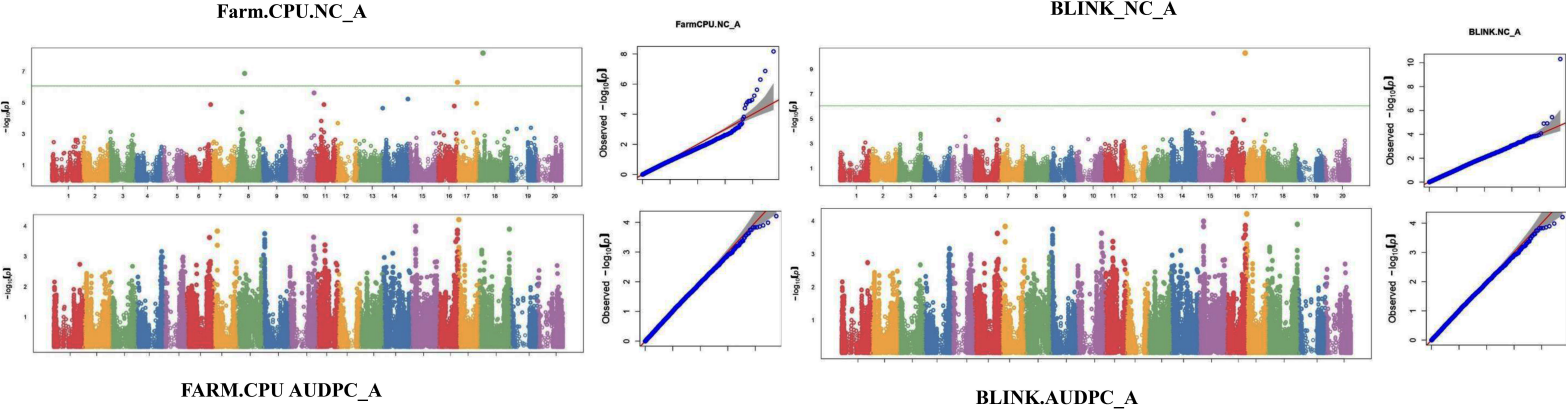
Manhattan plots (left) and quantile–quantile (Q–Q plots) (right) of the genome-wide association results for RSS generated through BLINK in 2021, 2022, and 2023.

The GWAS study uncovered several SNPs associated with charcoal rot resistance traits. In the case of the glasshouse experiment, a total of eight SNPs were identified to be associated with charcoal rot resistance at the seedling stage (**Table 6** and **Figure 8**). Of them, one SNP each was located on chromosome 8 (*S8_16817767*), chromosome 10 (*S10_52066337*), chromosome 14 (*S14_50857981*), chromosome 15 (*S15_32620059*), chromosome 17 (*S17_1689021*), and chromosome 18 (*S18_9413708*). Two SNPs—*S16_34569104* and *S16_37878937*—were located on chromosome 16. In the case of the field experiment, 10 SNPs were found to be associated with charcoal rot resistance at the adult plant stage (**Table 7** and **Figures 9****-****11**). Of them, two SNPs each were located on chromosome 6 (*S6_41109641* and *S6_41863847*) and chromosome 10 (*S10_40644409* and *S10_44768495*). One SNP each was identified on chromosome 12 (*S12_14977708*), chromosome 14 (*S14_51718686*), and chromosome 16 (*S16_33491560*), while three SNPs were located on chromosome 18 (*S18_25004105*, *S18_55655188*, and *S18_56366541*). The SNP *S14_51754926*, which was identified through both models, with a high *p*-value and associated with multiple resistance traits was considered for haplotype analysis.

**Table 6:** SNPs associated with charcoal rot resistance under glasshouse conditions.

S. no.	SNP	Chr	Position (Williams 82)	Model	Trait	*p*-value	Effect	SIG/SUG
1	S8_16817767	8	16522710	FarmCPU	NLA	1.36E−07	0.3897	SUG
2	S10_52066337	10	48596702	FarmCPU	NLA	2.39E−06	8.0376	SUG
3	S14_50857981	14	46785684	FarmCPU	NLA	5.89E−06	0.9875	SUG
4	S15_32620059	15	35894370	Blink	NLA	3.65E−06	0.9875	SUG
5	S16_34569104	16	32934723	FarmCPU	NLA	1.64E−05	−0.6075	SUG
6	S16_37878937	16	36707683	Blink	NLA	1.24E−05	0.3256	SUG
7	S17_1689021	17	1665571	FarmCPU	AUDPCA	6.18E−05	14.0025	SUG
				Blink	AUDPCA	6.18E−05	287.9616	SUG
				Blink	NLA	4.89E−11	0.0814	SIG
				FarmCPU	NLA	5.04E−07	−0.9428	SUG
8	S18_9413708	18	9646515	FarmCPU	NLA	6.81E−09	1.0012	SIG

SIG, significant; SUG, suggestive.

**Table 7:** SNPs associated with charcoal rot resistance under sick plot conditions.

S. no.	SNP	Chr	Position (Williams 82)	Model	Trait	Year	*p*-value	Effect	SIG/SUG
1	S6_41109641	6	40867173	Blink	PDI	2021	2.74E−05	0.0775	SUG
				FarmCPU	PDI	2021	1.44E−07	−17.0573	SUG
				Blink	RSS	2023	6.00E−05	0.0860	SUG
				FarmCPU	RSS	2023	6.00E−05	0.4866	SUG
2	S6_41863847	6	41371875	Blink	PDI	2021	4.20E−05	0.0775	SUG
				Blink	AUDPC	2021	1.36E−07	0.1033	SUG
3	S10_40644409	10	37297350	FarmCPU	PDI	2021	1.18E−07	−17.0573	SUG
4	S10_44768495	10	41391571	FarmCPU	PDI	2021	5.03E−06	7.7626	SUG
5	S12_14977708	12	14725785	FarmCPU	PDI	2021	2.12E−06	−9.1799	SUG
6	S14_51754926	14	47666485	Blink	AUDPC	2021	2.64E−09	0.3897	SIG
				FarmCPU	AUDPC	2021	3.23E−05	251.4130	SUG
				Blink	PDI	2021	1.33E−09	0.3897	SIG
				FarmCPU	PDI	2021	7.05E−07	8.0376	SUG
7	S16_33491560	16	31867097	FarmCPU	PDI	2021	5.06E−06	7.7626	SUG
8	S18_25004105	18	25092187	Blink	AUDPC	2023	9.28E−06	0.1698	SUG
				FarmCPU	AUDPC	2023	9.28E−06	0.1698	SUG
				Blink	PDI	2023	3.44E−05	0.1698	SUG
				FarmCPU	PDI	2023	3.44E−05	0.1698	SUG
9	S18_55655188	18	52619040	Blink	PDI	2022	9.49E−05	0.2605	SUG
				FarmCPU	PDI	2022	9.49E−05	0.2605	SUG
10	S18_56366541	18	53324416	Blink	AUDPC	2022	7.30E−05	0.2628	SUG
				FarmCPU	AUDPC	2022	7.30E−05	0.2628	SUG
				Blink	PDI	2022	7.14E−05	0.2605	SUG
				FarmCPU	PDI	2022	7.14E−05	0.2605	SUG

SIG, significant; SUG, suggestive.

### 3.6 The putative candidate genes analysis

The putative candidate genes analysis was performed for six loci detected for multiple resistance traits. Within 400-kb genomic regions of these loci, a total of 23 genes with annotations associated with defense response pathway were identified (**Table 8**). Under glasshouse conditions, 5 genes (*Glyma.14G203000*, *Glyma.14G203700*, *Glyma.14G204500*, *Glyma.14G204600*, and *Glyma.14G205000*) were identified within the region of *SNP S14_50857981*, while 3 genes (*Glyma.17G021400*, *Glyma.17G022700*, and *Glyma.17G023400*) were identified within the region of *S17_1689021*, whereas under field conditions, 13 genes involved in defense response were identified. On chromosome 6, a putative candidate gene, *Glyma.06G244200*, was identified in the region of SNP *S6_41109641*. Six genes, *Glyma.14G209900*, *Glyma.14G210200*, *Glyma.14G211300*, *Glyma.14G211600*, *Glyma.14G212200*, and *Glyma.14G212500*, were identified on chromosome 14, near the peak SNP *S14_51754926*. Furthermore, on chromosome 18, five genes (*Glyma.18G236800*, *Glyma.18G237900*, *Glyma.18G238700*, *Glyma.18G239600*, and *Glyma.18G239700*) were identified within the region of *S18_55655188* while three genes (*Glyma.18G245900, Glyma.18G246400*, and *Glyma.18G248100*) were identified near *SNP S18_56366541*. Details of the gene models and their biological functions are given in **Table 8**.

**Table 8:** Candidate genes with biological process description and PFAM descriptions.

Experiment	Loci	Genes	Start	Stop	Biological process, description	PFAM_descriptions
Glasshouse	S14_50857981	Glyma.14G203000	46787303	46790554	Abscisic acid, jasmonic acid, and ethylene-mediated signaling pathway	NAF domain; protein kinase domain
		Glyma.14G203700	46828582	46834138	Cellular response to water deprivation; galactolipid biosynthetic process; organ senescence; protein autophosphorylation	Protein kinase domain
		Glyma.14G204500	46946496	46957734	Defense response	NB-ARC domain
		Glyma.14G204600	46968705	46974585	Defense response	NB-ARC domain
		Glyma.14G205000	47005574	47019661	Defense response	NB-ARC domain
	S17_1689021	Glyma.17G021400	1566684	1575389	Response to jasmonic acid stimulus; response to wounding	Inosine–uridine preferring nucleoside hydrolase
		Glyma.17G022700	1661386	1663779	Cellular response to osmotic stress; response to fungus	F-box domain; Tub family
		Glyma.17G023400	1703497	1707050	Negative regulation of defense response to bacterium; protein ubiquitination	CHY zinc finger; zinc finger, C3HC4 type (RING finger)
Field	S6_41109641	Glyma.06G244200	40759629	40760516	Abscisic acid and ethylene-mediated signaling pathway; intracellular signal transduction; protein phosphorylation; response to water deprivation	Protein kinase domain
	S14_51754926	Glyma.14G209900	47515899	47521687	Callose deposition in phloem sieve plate; galactolipid biosynthetic process; sucrose biosynthetic process	Sucrose synthase; glycosyl transferases group 1
		Glyma.14G210200	47535839	47545840	Autophagy; defense response to fungus; leaf senescence; response to starvation	Autophagy protein Apg5
		Glyma.14G211300	47625904	47630956	Defense response to fungus	Universal stress protein family
		Glyma.14G211600	47645652	47651977	RNA processing; nuclear-transcribed mRNA catabolic process; response to salt stress	PRP38 family
		Glyma.14G212200	47742907	47744519	Intracellular signal transduction; proline transport; protein ubiquitination; response to chitin	U-box domain
		Glyma.14G212500	47758067	47768578	Defense response to fungus; response to abscisic acid stimulus; response to chitin; transmembrane transport	Ankyrin repeat; domain of unknown function (DUF3354); cyclic nucleotide-binding domain; ion transport protein
	S18_55655188	Glyma.18G236800	52562784	52568422	Abscisic acid, jasmonic acid and ethylene-mediated signaling pathway; defense response to fungus; detection of biotic stimulus; intracellular signal transduction	Protein kinase domain
		Glyma.18G237900	52656634	52660776	Protein phosphorylation; response to abscisic acid stimulus	Protein kinase domain; salt stress response/antifungal
		Glyma.18G238700	52756503	52759147	Defense response to fungus; response to water deprivation; response to wounding	Late embryogenesis abundant protein
		Glyma.18G239600	52837347	52840571	Defense response to fungus; protein autophosphorylation; response to chitin	LysM domain; protein kinase domain
		Glyma.18G239700	52842315	52844380	Defense response to fungus; protein autophosphorylation; response to chitin	Protein kinase domain; LysM domain
	S18_56366541	Glyma.18G245900	53353413	53355664	Defense response to virus; fatty acid biosynthetic process; production of miRNAs involved in gene silencing; production of ta-siRNAs involved in RNA interference	Phosphopantetheine attachment site
		Glyma.18G246400	53390263	53393453	Innate immune response; negative regulation of cell death; salicylic acid biosynthetic process; systemic acquired resistance	Leucine-rich repeat N-terminal domain
		Glyma.18G248100	53511479	53516777	Protein folding; response to oxidative stress	Cyclophilin type peptidyl-prolyl cis-trans isomerase/CLD

### 3.7 Haplotype analysis

**Figure 12:**
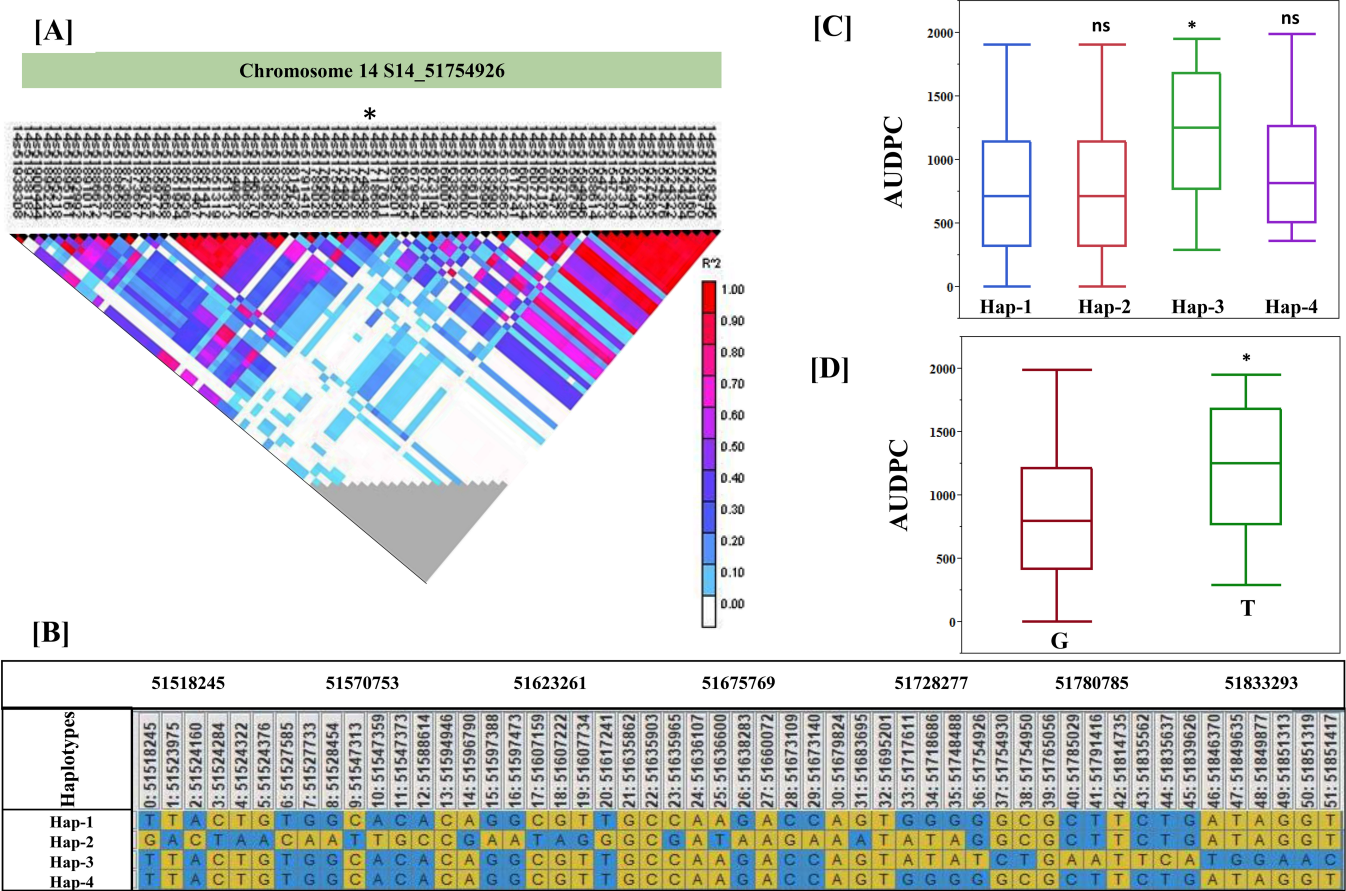
Haplotype analysis. **(A)** Significant SNPs on chromosome 14. **(B)** Four major haplotypes: Hap1, Hap2, Hap3, and Hap4. **(C)** Haplotype association with AUDPC. **(D)** Allelic effect on AUDPC. * - significance at p<0.05, ns - non-significance.

Haplotype analysis was performed on the most significant locus identified in this study, located on chromosome 14, carrying a peak SNP, *S14_51754926* (**Figure 12A**). The results identified four major haplotypes: Hap1, Hap2, Hap3, and Hap4 (**Figure 12B**). The data showed that Hap1 was significantly associated with a lower AUDPC, indicating a relation to charcoal resistance. In contrast, Hap3 was associated with susceptibility, as it exhibited higher AUDPC (**Figure 12C**). Furthermore, a significant difference was found in the mean of the AUDPC in two groups with allelic difference at the peak SNP, *S14_51754926* (**Figure 12D**).

## 4 Discussion

Charcoal rot in soybean is a devastating disease that can be a threat to soybean production and sustainability. Nevertheless, very few studies have been carried out in identifying potential resistance donors and gene or loci governing the resistance. Screening for charcoal rot resistance is based on both field conditions (Coser et al., 2017; Amrate et al., 2023, 2024) and glasshouse conditions (Coser et al., 2017; Amrate et al., 2024). There are a few reports on the identification of loci governing soybean charcoal rot resistance through GWAS and bi-parental mapping (Coser et al., 2017; Silva et al., 2019; Vinholes et al., 2019 and Zatybekov et al., 2023). Previous reports are based on a glasshouse experiment and/or a single season field data. To our knowledge, this is the first report on GWAS on soybean charcoal rot resistance based on the evaluation of multiple field trials and under controlled conditions. Such studies will minimize the effect of disease escape in identifying the true associations. For example, in the current study, an SNP, S6_41109641, was found to be associated with charcoal rot resistance in 2021 and 2023 through both models.

Furthermore, no SNP was found to be associated in both seedling and adult plant resistance, indicating different defense pathways being activated at different growth stages (Coser et al., 2017). This may further be justified by the fact that the environmental conditions for both glasshouse and field experiments were different and stress induced under field conditions was gradual, whereas that of artificial inoculation was through wounding, which was acute, resulting in differential gene expressions and pathways (Coser et al., 2017). While this is plausible, alternative causes such as limited statistical power or phenotype–environment interactions cannot be ruled out. However, SNP S14_50857981 identified in artificial conditions and S14_51754926 identified in field conditions are present within a 1-Mb region and may constitute a quantitative trait locus (QTL). Such QTL will be of immense importance in breeding for seedling and adult plant resistance.

A peak SNP Gm16_36809255 was reported to be linked to charcoal rot seedling resistance in soybean (Silva et al., 2019). In our study, an SNP, S16_37878937 (position in Williams 82-36707683), which was in proximity with this reported SNP, was found to be associated with the resistance at the seedling stage. Such genomic regions should be focused for allele and gene mining for charcoal rot resistance. Furthermore, the present study identified several previously unreported novel resistance sources, which can be used for validation with the aim of using them in breeding programs for durable resistance against charcoal rot.

In addition to the identification of suggestive and significant SNPs, haplotype analysis can provide novel insights into the genetic determinants of trait (Vinholes et al., 2019). In our current study, the identified haplotype can be used in haplotype breeding for resistance against charcoal rot resistance. These haplotypes are associated with defense responsive genes—*Glyma.14G209900* (callose deposition, galactolipid, and sucrose biosynthesis), *Glyma.14g210200* (involved in autophagy), *Glyma.14g211300* (universal stress protein), *Glyma.14G211600* (PRP38 family), *Glyma.14g212200* (signal transduction), and *Glyma.14g212500* (PAS/PAC sensor domain). Such genes, after validation, can be used in genome editing experiments to improve charcoal rot resistance in soybean.

In case of cowpea [*Vigna unguiculata* (L) Walp.], loci conferring charcoal rot resistance were co-localized with those of drought tolerance and there was a correspondence between *M. phaseolina* resistance haplotypes and drought tolerance haplotypes. Furthermore, soybean genomic regions harboring genes responsive for heat shock, sodium hypersensitivity, and calcium sensing were syntenic to the charcoal rot resistance loci identified in cowpea (Muchero et al., 2011). Late embryogenesis abundant (LEA) proteins are attributed to the plant defense against drought stress (Chen et al., 2021). Two LEA protein-coding genes (*Glyma_19G198800* and *Glyma_19G198900*) were reported to be involved in charcoal rot resistance in soybean (Zatybekov et al., 2023). Similarly, in our study, an LEA protein-coding gene, *Glyma.18g238700*, was found to be present in the locus associated with charcoal rot resistance. Such genes can be investigated for their possible role in drought tolerance and charcoal rot.

Furthermore, leucine-rich repeat receptor-like protein kinases were reported to be involved in resistance mechanism against *M. phaseolina* in sesame (*Sesamum indicum*) (Yan et al., 2021). We found three leucine rich repeat receptor-like protein kinase encoding genes (Glyma.17g019800, Glyma.06g244100 and Glyma.18g240800) associated with charcoal rot resistance. Abscisic acid (ABA), salicylic acid (SA), jasmonic acid (JA), and ethylene were reported to be involved in the defense mechanism against different diseases in plants (Anderson et al., 2004). In the current study, several putative candidate genes involved in the ABA-mediated pathway (*Glyma.14G203000, Glyma.06G244200, Glyma.14G212500*, and *Glyma.18G236800*), SA-mediated pathway (*Glyma.18G246400*), JA-mediated pathway (*Glyma.14G203000, Glyma.17G021400*, and *Glyma.18G236800*), and ethylene-mediated pathway (*Glyma.14G203000, Glyma.06G244200*, and *Glyma.18G236800*) were identified as candidate genes for charcoal rot resistance. A cyclophilin protein-encoding gene, *Glyma.18G248100*, was found to be associated with the charcoal rot resistance under field conditions. The same gene was also previously reported to be associated with field resistance against charcoal rot disease (Coser et al., 2017).

The identification of potential resistance donors is crucial in any disease resistance breeding program. The soybean introduction, PI 159923, was found to be resistant under both field and glasshouse conditions, and such genotypes are of immense importance in deploying charcoal rot resistance in cultivars. Furthermore, this genotype was previously reported to be resistant against purple seed stain (Alloatti et al., 2015) and high SMR (stem reserve mobilization) (Satpute et al., 2020). The genotype AGS 25 identified in the current study was previously reported to carry long juvenility (Gupta et al., 2021) and such genotypes would aid in the development of wider adaptable charcoal rot-resistant genotypes/varieties. In addition, charcoal rot-resistant genotypes JS 20–76 and EC 602288 were previously reported to be water logging tolerant (Chandra et al., 2023). These genotypes will be used in breeding for multiple stress-tolerant varieties.

## 5 Conclusion

In the present study, 8 SNPs linked to charcoal rot resistance at the seedling stage were identified, while 10 SNPs were found to be associated with adult plant resistance. Haplotype analysis of SNP *S14_51754926* revealed that out of four haplotypes, Hap1 was significantly associated with lower AUDPC, indicating a relation to charcoal resistance. The putative candidate gene analysis in genomic regions of significant SNPs identified 23 genes, with annotations associated with defense response and antifungal activity, and involved in the signaling pathway. In addition, PI 159923 was found to be resistant under both field and glasshouse conditions; such a genotype will be employed as a parent in breeding for high-yielding charcoal rot-resistant genotypes.

## Data Availability

The original contributions presented in the study are publicly available. This data can be found here: NCBI, PRJNA1367327.
